# Supplementary figures and images for: Pre-exposure to mRNA-LNP inhibits adaptive immune responses and alters innate immune fitness in an inheritable fashion
Source: bioRxiv. 2022 Aug 20:2022.03.16.484616. Preprint. [Version 2] doi: 10.1101/2022.03.16.484616 (PMC9413714; doi:10.1101/2022.03.16.484616)

Suppl. Figure 1

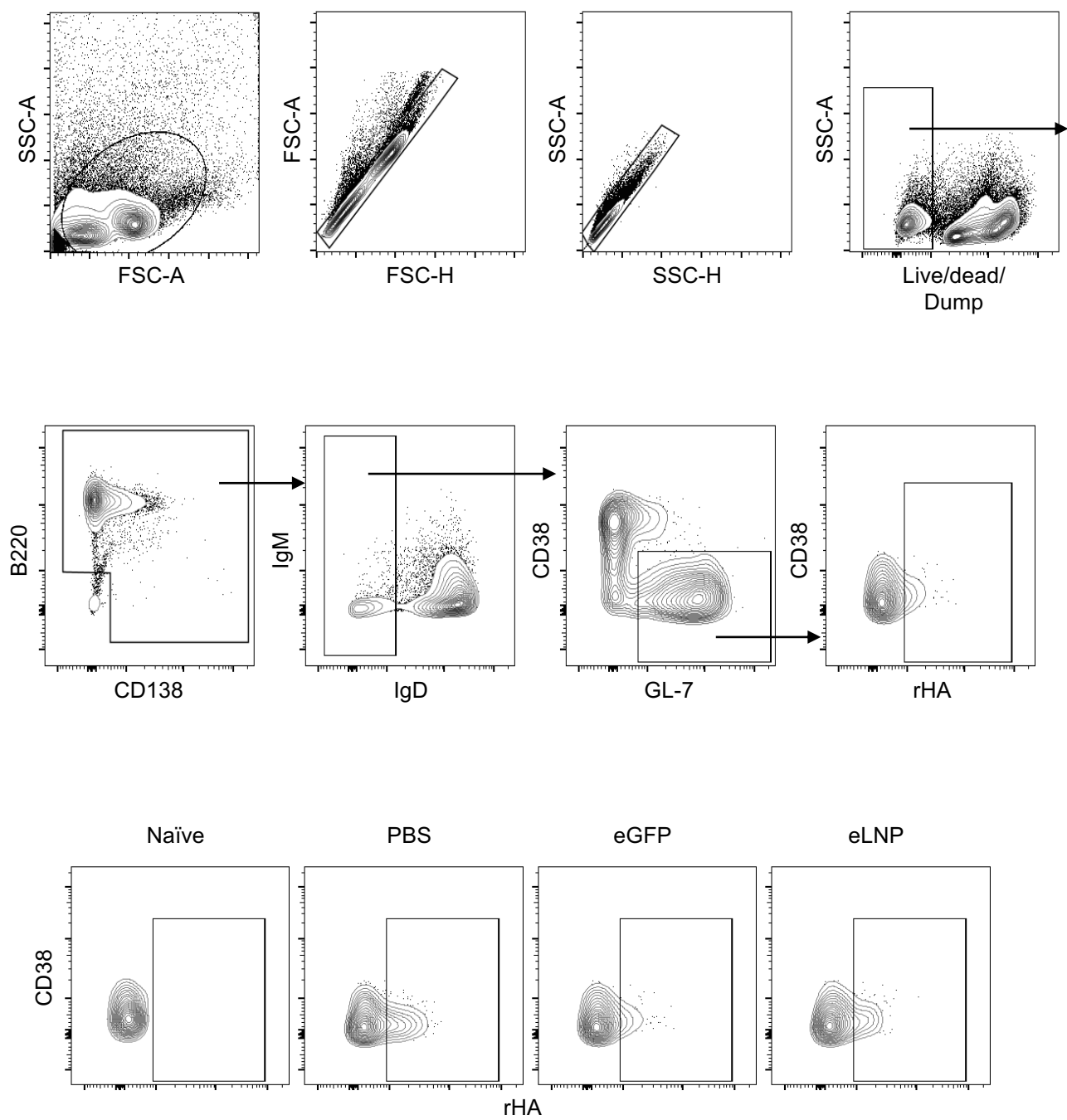

Suppl. Figure 2

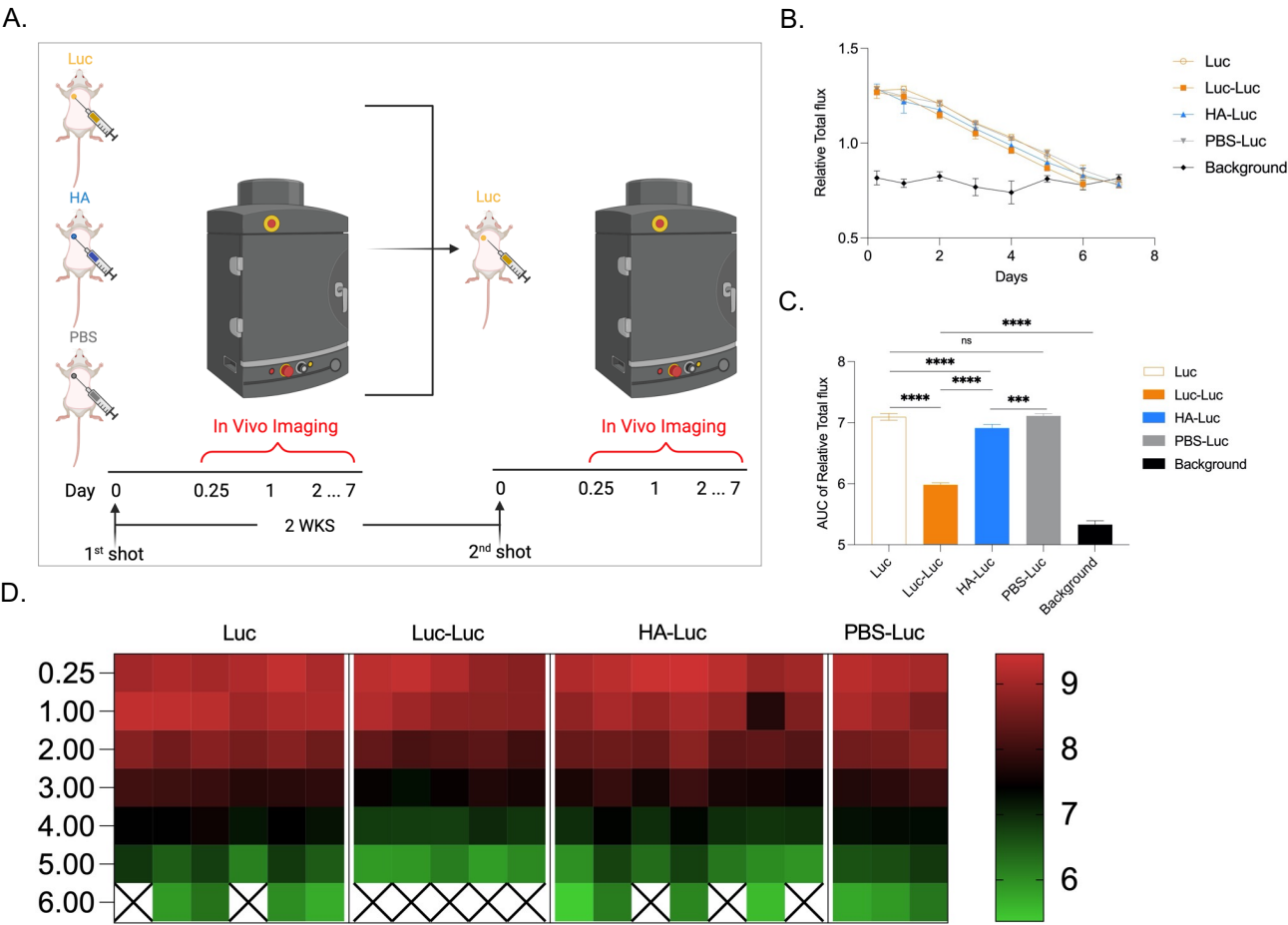

Suppl. Figure 3

A.

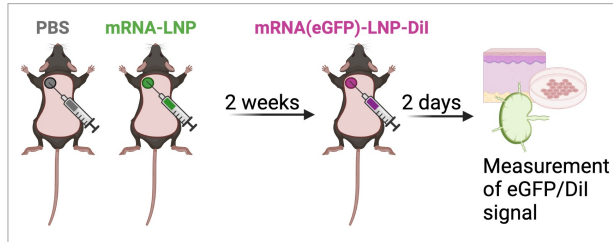

B.

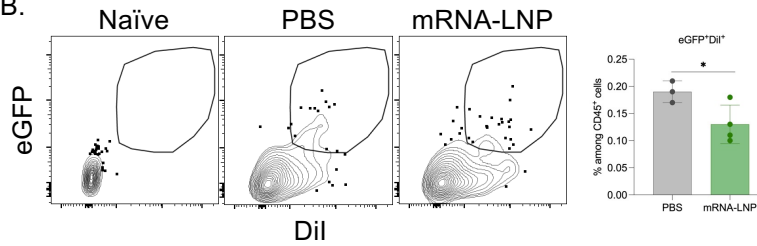

C.

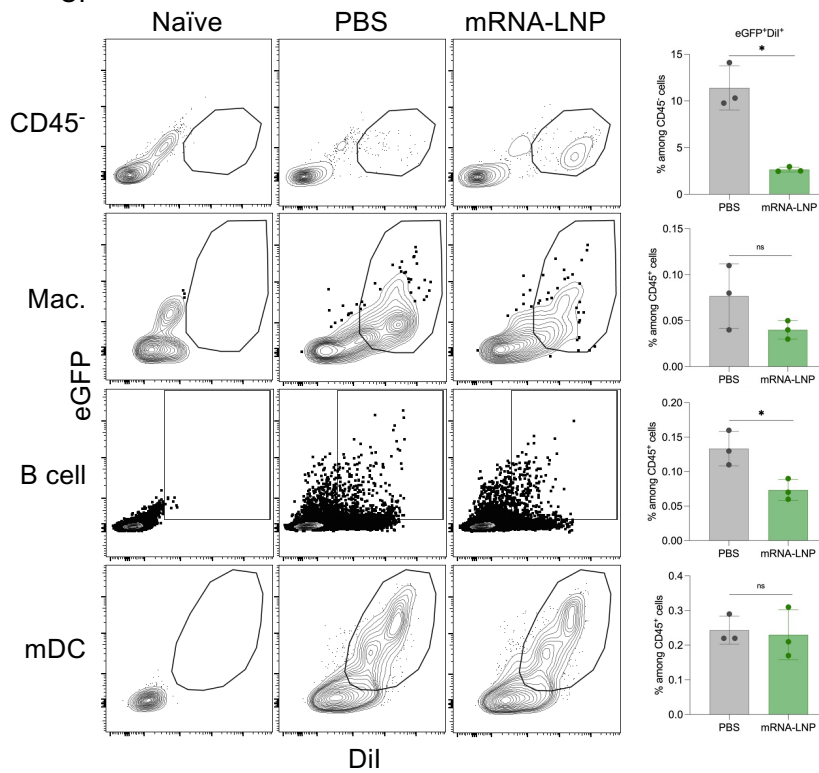

Suppl. Figure 4

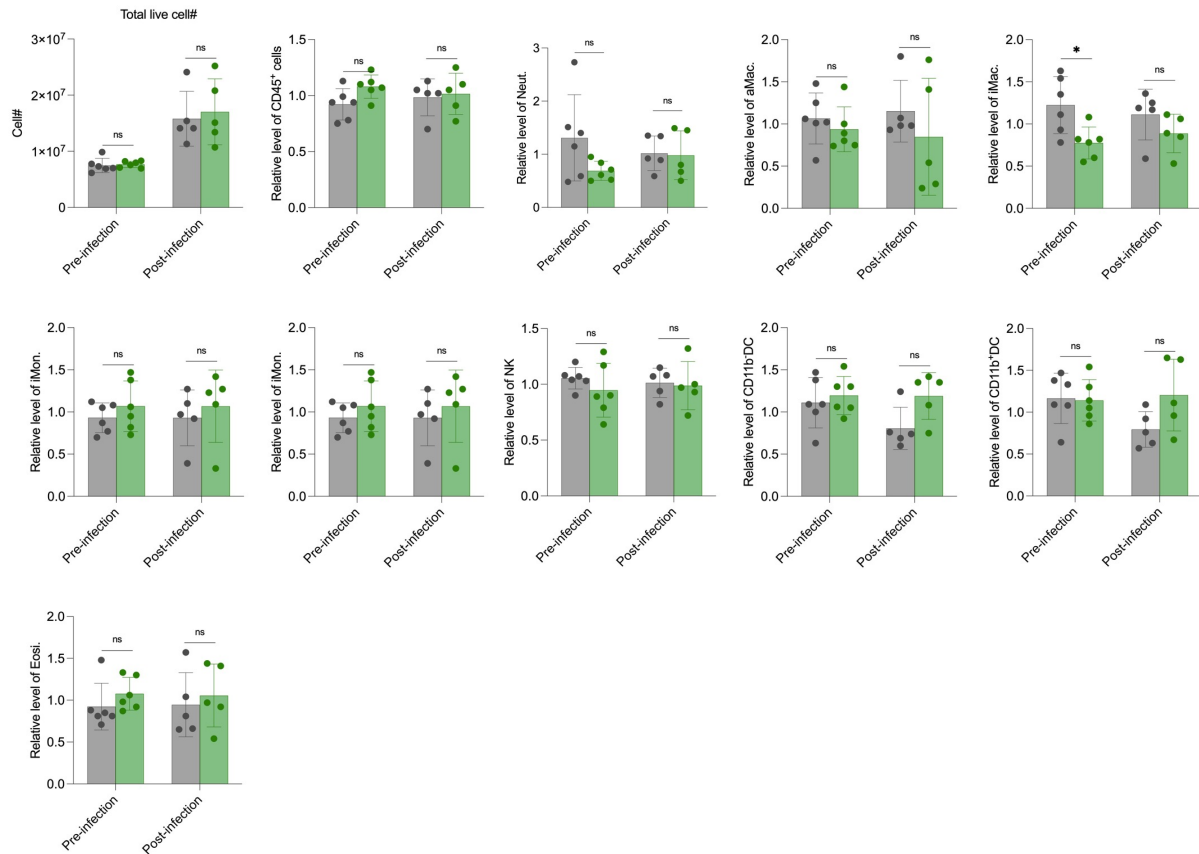

Suppl. Figure 5

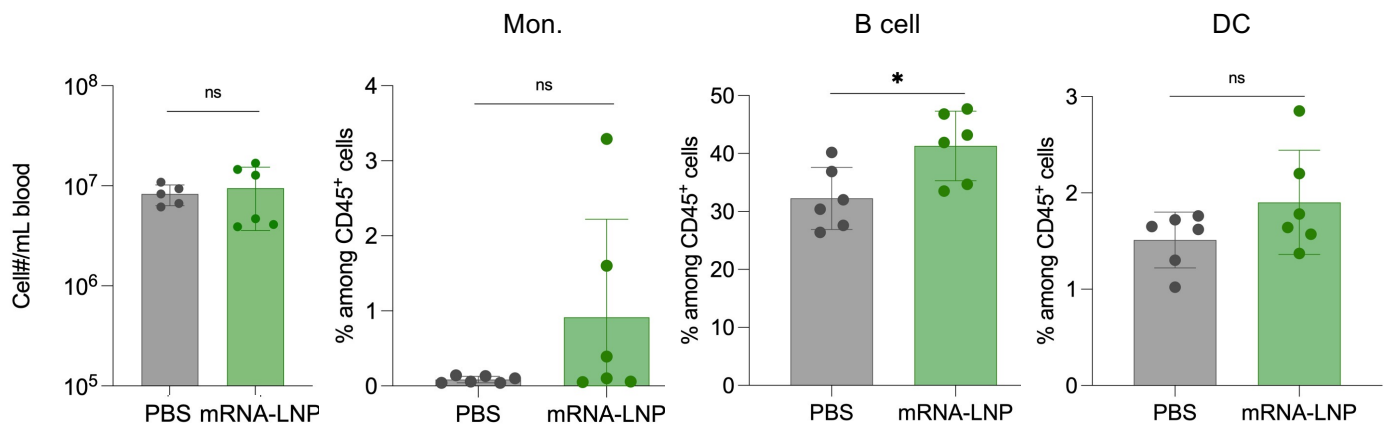

Suppl. Figure 6

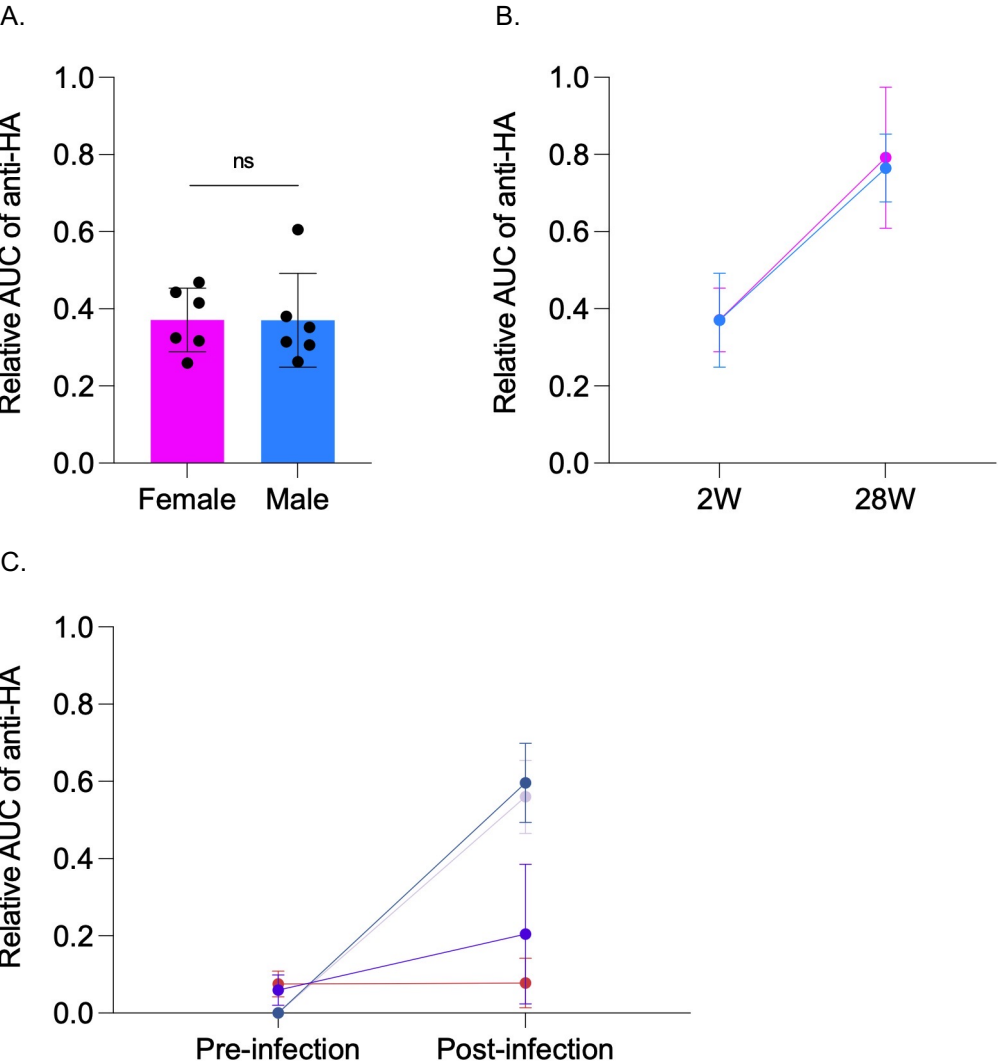

Supplement: Supplement 1 — Supplemental Figure 1. Gating strategy for HA-specific GC B cells Supplemental Figure 2. Pre-exposure to mRNA-LNPs decreases antigen levels. A). Experimental model. Balb/c mice were pre-exposed to PBS, Luc mRNA-LNPs or PR8 HA mRNA-LNPs and imaged using IVIS 6 hours (0.25 day) post inoculation and then every day for 7 days. Two weeks later all the animals were injected in the same spot with Luc mRNA-LNPs and the luciferase signal monitored similarly to the first exposure. B). Relative total flux with time. C). Data from B presented as AUC. D). Total flux values (background subtracted) of each mouse at different time points are shown as log10. X marks mice where the signal was below detection. Data from two separate experiments pooled. One way ANOVA was used to establish significance. ns = not significant. ***p<0.0005, ****p<0.0001. Supplemental Figure 3. Pre-exposure to mRNA-LNPs leads to overall decrease of antigen levels. A). Experimental model. Animals were shaved and intradermally inoculated in the left upper spot with either PBS or mRNA-LNP coding for HA. Two weeks later the same areas were injected with mRNA-LNP-DiI coding for eGFP. The injected skin (2cm2) and skin draining lymph nodes were harvested 2 days later and the eGFP and DiI signals determined using flow cytometer. B). Representative flow plots and summary graph on eGFP+DiI+ population of skin DCs (MHCII+ CD11c+) after gating on live cell/Ly-6G−/CD64−. Naïve mice were not injected with mRNA-LNP-DiI coding for eGFP. C). Representative flow plots and summary graphs on eGFP+DiI+ population of SDLNs CD45− cells, macrophages (Mac., CD64+), B cells (MHCII+CD11c−) and mDCs (MHCIIhigh CD11cmid). Each dot represents a separate mouse. The data are from one experiment and are shown as mean ±SD. Welch’s t test was used to establish significance. ns = not significant. *p<0.05. Supplemental Figure 4. Characterization of lung immune cells before and after influenza challenge in PBS or mRNA-LNP pre-exposed mice. Summ [file media-1.pdf]
